# Long-term expansion of human hematopoietic stem cells

**DOI:** 10.1186/s13619-023-00163-w

**Published:** 2023-04-19

**Authors:** Guixian Liang, Feng Liu

**Affiliations:** 1 State Key Laboratory of Membrane Biology, Institute of Zoology, Institute for Stem Cell and Regeneration, Chinese Academy of Sciences, University of Chinese Academy of Sciences, Beijing, 100101 China; 2 Institute for Stem Cell and Regeneration, Chinese Academy of Sciences, Beijing, 100101 China; 3 School of Life Sciences, Shandong University, Qingdao, 266237 China

## Abstract

Hematopoietic stem cells (HSCs) are critical for the treatment of a variety of hematological diseases. However, the low number of HSCs lead to the clinical application difficult. To gain more functional human HSCs ex vivo, Sakurai et al. established a recombinant-cytokine-free and albumin-free culture system, i.e. PCL-PVAc-PEG-based culture, in combination with 740Y-P, butyzamide and UM171, to improve the long-term expansion of human cord blood HSCs.

## Main text

In vertebrates, hematopoiesis occurs in different sites, including the yolk sac, aorta-gonad-mesonephros (AGM), placenta and fetal liver in mammals, and the AGM and caudal hematopoietic tissue in zebrafish (Gao et al. 2022; Liang et al. 2021; Xia et al. 2021). Hematopoietic stem cells (HSCs) emerge during embryogenesis and have the ability of self-renewal and differentiation to support life-long hematopoiesis (Dzierzak and Bigas 2018). Hematopoietic stem cell transplantation (HSCT) is a critical treatment for a variety of hematological diseases and HSC is the functional unit of HSCT (Rowe et al. 2016). In clinic, HSCs can be collected from human umbilical cord blood, but the number of HSCs in cord blood is limited. Many previous studies made efforts to promote the expansion of functional HSCs, usually using recombinant cytokines combined with serum albumin (Wilkinson et al. 2020). Nevertheless, the cytokine and albumin culture system can only support the short-term maintenance of HSCs, therefore a system, which can promote the long-term expansion of functional HSCs needs to be established.

### Chemical substitution of cytokines in the culture system

In their previous study, Wilkinson et al. established a long-term ex vivo expansion system for functional mouse HSCs using recombinant stem cell factor (SCF), thrombopoietin (THPO), and synthetic polymer polyvinyl alcohol (PVA) (Wilkinson et al. 2019). In this work (Sakurai et al. 2023), firstly, the authors used the same culture system to expand CD34^+^CD38^−^ hematopoietic stem and progenitor cells (HSPCs) from human umbilical cord blood. The results showed that though mouse bone marrow Lineage^−^Sca-1^+^Kit^+^ (LSK) HSPCs expanded by around 18-fold in 7 days, whereas human HSPCs only expanded by around 3- to 4-fold during the same time. Based on these results, the authors suggested that the expansion system for functional mouse HSCs was not suitable for human cord blood HSCs, and the expansion culture system suitable for human cord blood HSCs needs to be established. To this end, the authors improved the expansion of human HSPCs by replacing recombinant cytokines with chemicals. They found that recombinant SCF and THPO can be replaced with a PI3K activator (740Y-P) and THPO-receptor agonist (butyzamide), respectively. However, the xenotransplantation assays showed that the supplements with 740Y-P and butyzamide only supported human HSCs in the short term, while they were not sufficient to stabilize the longer-term expansion of human HSCs.

### Long-term expansion of human functional HSCs

**Fig. 1:**
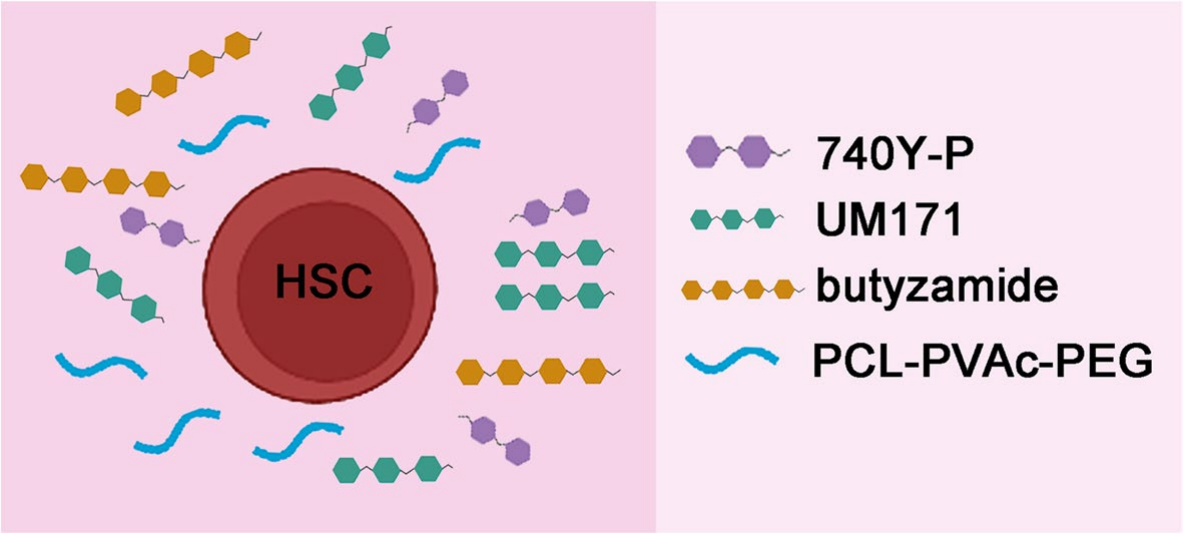
Components of ex vivo human HSC culture system. PCL-PVAc-PEG-based culture condition, in combination with a PI3K activator (740Y-P), THPO-receptor agonist (butyzamide) and UM171 can promote the long-term expansion of functional human cord blood HSCs ex vivo

To stabilize long-term ex vivo HSC expansion, the authors found that UM171, which has been reported as a HSPC expansion compound, can promote stable expansion of human HSCs for at least 1 month (Fares et al. 2014). Moreover, to increase the growth of HSCs, the authors screened ten polymers and identified Soluplus, a polyvinyl caprolactam-polyvinyl acetate-polyethylene glycol graft copolymer (PCL-PVAc-PEG), which can replace PVA. The PCL-PVAc-PEG-based culture condition, in combination with 740Y-P, butyzamide and UM171 (3a medium), can promote the long-term expansion, with an approximately 55-fold expansion of CD34^+^ cells after a 30-day culture. Transplantation assays showed that robust human cell chimerism was observed in the peripheral blood of xenotransplanted recipient mice, including first and secondary xenotransplantation. Collectively, these results suggest that PCL-PVAc-PEG-based 3a medium can support the robust expansion of functional human HSCs ex vivo (Fig. 1).

### Characterization of expanded human HSCs

To characterize the expanded human HSCs at the molecular level, the authors performed bulk RNA sequencing (RNA-seq) on CD34^high^EPCR^+^ and CD34^high^EPCR^−^ cells from 10-day culture in PCL-PVAc-PEG-based 3a medium. Transcriptomic analysis showed that LT-HSC markers *HLF* and *AVP* were highly expressed in the CD34^high^EPCR^+^ population. Moreover, gene set enrichment analysis (GSEA) showed that HSC gene sets and genes related to the lysosomal membrane were enriched in the CD34^high^EPCR^+^ population, consistent with the previous report (Garcia-Prat et al. 2021). Furthermore, to investigate the heterogeneity within the expanded human HSCs, the authors performed single-cell RNA-seq using three culture condition, including PCL-PVAc-PEG-based 3a medium, and Stem-Span SFEM supplemented with SR-1 or UM171. After integration and analysis, the authors identified and annotated 12 major clusters, including 3 HSPC clusters and 9 progenitor clusters. Among 3 HSPC clusters, HSC genes *HLF* and *AVP* were highly expressed in HSPC-HLF cluster, and lowly expressed in HSPC and HSPC-cycling clusters. Comparing the cellular composition of three culture conditions, the authors found that PCL-PVAc-PEG-based 3a medium was more suitable for the expansion of HSPC-HLF cluster, which they considered to be long-term HSCs.

### Clonal expansion of human HSCs

To examine whether PCL-PVAc-PEG-based 3a medium could support the expansion of clonally derived HSC cultures, the authors sorted single cord-blood-derived CD34^+^CD38^−^CD90^+^CD45RA^−^CD49f^+^ cell into 96-well plates and cultured with PCL-PVAc-PEG-based 3a medium. After 7 days, the heterogeneous clonal expansion was observed, implying that the cord-blood-derived CD34^+^CD38^−^CD90^+^CD45RA^−^CD49f^+^ cell is heterogenous. Moreover, single HSC-derived cultures that exhibited high expansion rate were transplanted into recipient mice. The results showed that half of recipients exhibited robust chimerism in the peripheral blood, bone marrow and spleen, suggesting that PCL-PVAc-PEG-based 3a medium can support clonal expansion of human HSCs ex vivo.

## Conclusions

In summary, Sakurai et al. developed a recombinant-cytokine-free and albumin-free culture system that expands functional human HSCs. PCL-PVAc-PEG-based 3a medium may be advantageous, because it can facilitate rapid expansion, improve expansion stability and reduce reagent costs. This culture system has the potential clinical implications for solving the shortage of donor HSCs in allogeneic HSC transplantation.

## Data Availability

Not applicable.
